# Acidic Nanoparticles Are Trafficked to Lysosomes and Restore an Acidic Lysosomal pH and Degradative Function to Compromised ARPE-19 Cells

**DOI:** 10.1371/journal.pone.0049635

**Published:** 2012-12-18

**Authors:** Gabriel C. Baltazar, Sonia Guha, Wennan Lu, Jason Lim, Kathleen Boesze-Battaglia, Alan M. Laties, Puneet Tyagi, Uday B. Kompella, Claire H. Mitchell

**Affiliations:** 1 Department of Anatomy and Cell Biology, University of Pennsylvania, Philadelphia, Pennsylvania, United States of America; 2 Department of Biochemistry, University of Pennsylvania, Philadelphia, Pennsylvania, United States of America; 3 Department of Ophthalmology, University of Pennsylvania, Philadelphia, Pennsylvania, United States of America; 4 Department of Physiology, University of Pennsylvania, Philadelphia, Pennsylvania, United States of America; 5 Pharmaceutical Sciences and Ophthalmology, University of Colorado Denver, Aurora, Colorado, United States of America; University of Florida, United States of America

## Abstract

Lysosomal enzymes function optimally in acidic environments, and elevation of lysosomal pH can impede their ability to degrade material delivered to lysosomes through autophagy or phagocytosis. We hypothesize that abnormal lysosomal pH is a key aspect in diseases of accumulation and that restoring lysosomal pH will improve cell function. The propensity of nanoparticles to end up in the lysosome makes them an ideal method of delivering drugs to lysosomes. This study asked whether acidic nanoparticles could traffic to lysosomes, lower lysosomal pH and enhance lysosomal degradation by the cultured human retinal pigmented epithelial cell line ARPE-19. Acidic nanoparticles composed of poly (DL-lactide-co-glycolide) (PLGA) 502 H, PLGA 503 H and poly (DL-lactide) (PLA) colocalized to lysosomes of ARPE-19 cells within 60 min. PLGA 503 H and PLA lowered lysosomal pH in cells compromised by the alkalinizing agent chloroquine when measured 1 hr. after treatment, with acidification still observed 12 days later. PLA enhanced binding of Bodipy-pepstatin-A to the active site of cathepsin D in compromised cells. PLA also reduced the cellular levels of opsin and the lipofuscin-like autofluorescence associated with photoreceptor outer segments. These observations suggest the acidification produced by the nanoparticles was functionally effective. In summary, acid nanoparticles lead to a rapid and sustained lowering of lysosomal pH and improved degradative activity.

## Introduction

While the propensity of nanoparticles to accumulate in lysosomes can frustrate many, they are ideally suited to treat lysosomal defects. In this regard, the lysosomes of retinal pigment epithelial (RPE) cells represent a prime target. RPE lysosomes have a high degradative load, processing both the phagocytosed tips of shed photoreceptor outer segments and considerable autophagic material [3], [4]. The degradative lysosomal enzymes function optimally at an acidic pH; consequently, elevation of this pH is predicted to slow enzyme activity and decrease degradation. Lysosomal pH is elevated in RPE, as well as other cells, by basic drugs such as chloroquine and tamoxifen [5]–[7]. In addition, RPE lysosomes are alkalinized with delay by the bisretinoid N-retinylidene-N-retinylethanolamine (A2E) [8], and lysosomes of RPE from ABCA4^−/−^ mice having excess A2E are more alkaline than age matched controls [5]. The elevated pH can impair the activity of hydrolytic enzymes such as cathepsin D and lysosomal acid lipase, leading to a decline in lysosomal degradative capacity [9]–[11]. Incomplete degradation of phagocytic and autophagic material leads to the accumulation of autofluorescent lipofuscin which itself is often associated with retinal degenerations [12]–[14].

Treatment to restore an acidic lysosomal pH would be of therapeutic interest. Receptor-mediated pharmacologic intervention has demonstrated promise in lowering lysosomal pH and restoring the degradative capacity [5], [15], [16]. However, treatment that targets lysosomal acidity more directly may be advantageous. In this regard, polymeric nanoparticles (NPs) may be ideal. Nanoparticles prepared from biodegradable polymers are considered an attractive means of drug and gene delivery due to their non-toxic nature and their ability to be internalized into mammalian cells [17]. In the eye they have been utilized to deliver markers to mouse retinal neurons and genes into ARPE-19 cells [18]–[20]. When injected into the rabbit eye, magnetic nanoparticles were taken up by RPE cells and caused no discernable inflammation [21], while delivery of genetic material to the posterior eye can stop choroidal neovascularization following laser treatment in rodents [22]. Nanoparticles made from acidic PLGA nanoparticles are readily taken up into cells via endo-lysosomal phagocytotic pathways, implying they may be delivered to both the appropriate cell and organelle [23], [24]. We therefore asked if acidic nanoparticles derived from lactide and glycolide polymers could acidify the lysosomes of ARPE-19 cells to prevent the accumulation of autofluorescent material.

## Materials and Methods

### Ethics Statement

The use of bovine photoreceptor outer segments was approved by the University of Pennsylvania IACUC.

### Materials

Poly (DL-lactide-co-glycolide) (PLGA) Resomer® RG 502 H, PLGA Resomer® RG 503 H and poly (DL-lactide) (PLA) Resomer® R 203S were purchased from Boerhinger Ingelheim Inc., VA. Other material was purchased from Sigma Aldrich (MO) unless otherwise indicated.

### Cell Culture

The human ARPE-19 cell line was obtained from the American Type Culture Collection (Manassas, VA). [25] Cells were grown to confluence in 25 cm^2^ primary culture flasks in a 1∶1 mixture of Dulbecco’s modified Eagle medium (DMEM) and Ham’s F12 medium with 3 mM L-Glutamine, 100 µg/ml penicillin/streptomycin, 2.5 mg/ml Fungizone, and 10% fetal bovine serum (all Invitrogen Corp).

### Preparation and Storage of Nanoparticles

Three formulations of polymeric nanoparticles were developed by the lab of Dr. Uday Kompella; Nanoparticle 1 (NP1): PLGA 502 H; NP2: PLGA 503 H; and NP3 PLA. See Table 1. Nanoparticles were labeled with Nile Red for localization experiments (NP1R, NP2R and NP3R in Table 1). Unlabeled nanoparticles were used for measurements of lysosomal pH. All NPs were synthesized using the same process. Briefly, the polymer solution was prepared by dissolving the polymer in dichloromethane and transferring this to an aqueous solution of polyvinyl alcohol and sonicated (Misonix Sonicator 3000, Misonix Inc., NY) for 1 minute at an energy input of 10 W. The primary emulsion thus formed was further transferred to a larger volume of aqueous solution of polyvinyl alcohol and sonicated using a probe sonicator for 30 seconds at an energy input of 3 W. This step results in hardening of nanoparticles. The secondary emulsion was kept on stirring at room temperature for 3 hours to evaporate the organic solvent present in the nanoparticles. After 3 hours, the nanoparticles were harvested by centrifugation at 27000 g for 30 minutes. In order to remove the residual amount of polyvinyl alcohol present, the nanoparticles were washed twice by dispersing into water each time followed by centrifugation. The final nanoparticles pellet obtained after two washings was suspended in water and frozen at −80°C. The frozen nanoparticle dispersion was subjected to freeze drying overnight in a lyophilizer (Labconco Corporation, MO) to attain lyophilized nanoparticles. Nanoparticles were evaluated for mean particle size and polydispersity index (variance) using Nicomp 380 ZLS® Particle Sizer (Particle Sizing Systems, CA). One mg of nanoparticles were uniformly dispersed in 2 ml water and subjected to analysis. For experiments with ARPE-19 cells, all particles were desiccated at room temperature until reconstitution for same-day use.

**Table 1:** Physical properties of the polymers used in the study.

NP No	Nanoparticle	Polymer	MolecularWeight (Da)	Acid number(mg KOH/g)	Mean Particle size (nm)	Polydispersity index
1	Blank Nanoparticles	PLGA Resomer® RG 502 H	8000–10000	>6	503.9	0.321
2		PLGA Resomer® RG 503 H	30000–35000	>3	387.4	0.277
3		PLA Resomer®R 203 S	40000	∼ 1	428.5	0.240
1R	Nanoparticlesloaded with Nile red	PLGA Resomer® RG 502H	8000–10000	>6	383.5	0.237
2R		PLGA Resomer® RG 503H	30000–35000	>3	379.1	0.210
3R		PLA Resomer®R 203S	40000	∼ 1	397.8	0.218

### Visualization of Ingested Nanoparticles

**Figure 1:**
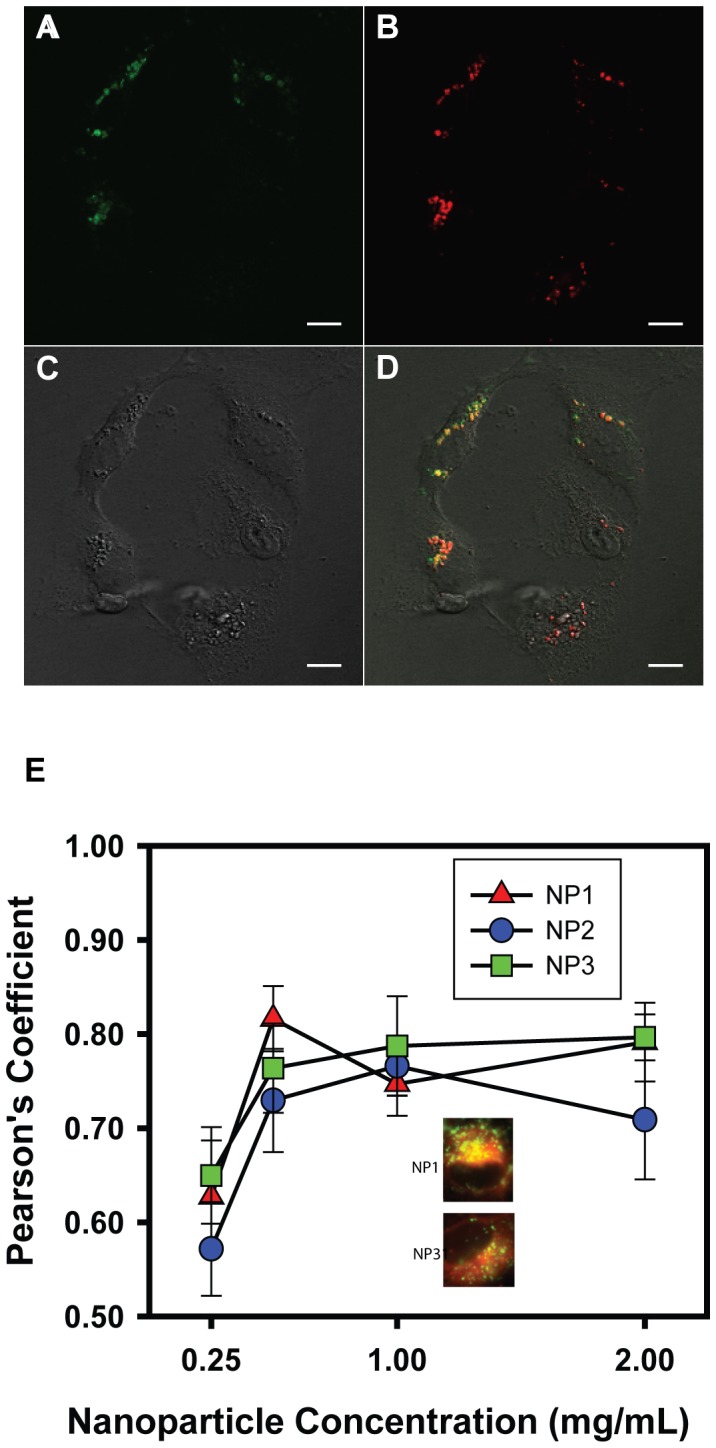
Acid nanoparticles are delivered to lysosomes of ARPE-19 cells. A-D. Live cell images of colocalized nanoparticles with ARPE-19 lysosomes after a 24 hour incubation with NP2R A. ARPE-19 cell lysosomes as visualized with the dye LysoTracker Green. Images taken at 40x magnification with a Zeiss confocal microscope. LysoTracker detected at 488/500 nm (excitation/emission). B. ARPE-19 ingestion of Nile Red stained nanoparticle NP2R. Cells were incubated for 24 hours in full culture medium with 1 mg/ml concentration of NPs. Nanoparticles were detected at 540/580 (excitation/emission). Before incubation, the nanoparticle suspension was passed through a 0.8 µM syringe filter to remove clumped particles. After incubation period, cells were washed thoroughly with isotonic solution in an attempt to further remove clumps and any extracellular NPs. C. DIC image of the ARPE-19 cells and nanoparticles. With this image the morphology of the ARPE-19 cells are clearly visible. D. Composite image of the LysoTracker Green (lysosomes), Nile Red (nanoparticles), and DIC exposures. This image demonstrates the colocalization of the ingested nanoparticles with the ARPE-19 lysosomes. E. Concentration dependence of lysosomal delivery. The degree of colocalization of NP1R, NP2R, and NP3R with lysotracker green as a function of concentration. Each point is the mean +/− SEM of a calculated Pearson’s coefficient within one area of interest (AOI) in one microscope field; n = 3 fields. Inserts indicate overlap of NP1R and NP3R. In 1E, NP2R were incubated for 1 hr. before colocalization was determined.

For concentration-dependent uptake studies, ARPE-19 cells were plated on 12 mm cover glass until reaching 50% confluence. Solutions of each Nile Red nanoparticle formulation in culture medium were sonicated and filtered at 0.8 µm (Thermo Fisher Scientific Inc., Waltham, MA). Initial images in Fig. 1A were obtained after 24 hr. incubation utilizing a Zeiss confocal microscope and processing at the University of Pennsylvania School of Medicine Biomedical Imaging Center. For studies quantifying the effect of concentration, NPs were present at 0.25, 0.5, 1.0 and 2.0 mg/ml and cells were incubated for 1 hr. For time-dependent studies, cells were incubated in 1 mg/ml NP solution for the time indicated. After incubation, cells were washed 3x with isotonic solution (IS; (in mM) NaCl 105, KCl 5, HEPES Acid 6, Na HEPES 4, NaHCO_3_ 5, mannitol 60, glucose 5, MgCl_2_ 0.5, CaCl_2_ 1.3), then incubated in 5 µM LysoTracker Green DND-26 (Invitrogen Corp., Carlsbad, CA) for 15 min. Cells were washed again 3x with IS before visualization of Nile Red nanoparticles (540 nm ex) or lysosomes with LysoTracker Green (488 nm ex, Molecular Proves/Invitrogen). For concentration studies, images were captured with an Eclipse E600 fluorescent microscope (Nikon Inc.) and Retiga 2000R CCD monochromatic camera (QImaging, BC, Canada) with processing by ImagePro (MediaCybernetics Inc., Bethesda, MD). Pearson’s coefficient for overlap between Nile Red and LysoTracker regions was determined with one or two cells defined as an area of interest. For time-dependent assays, cells were processed with a Nikon A1R confocal microscope system at the University of Pennsylvania Live Cell Imaging Core with NIS-Elements software (Nikon Inc.) was used to calculate the Pearson’s correlation coefficient from a cell. The z-stack with the greatest Pearson’s coefficient in each field was used for the calculations.

### Lysosomal pH Measurements from ARPE-19 Cells

In brief, cells were grown to confluence on black-walled, clear-bottomed Costar® 96-well plates (Corning Inc., Corning, NY) and grown to 100% confluence. The medium was removed and replaced with various drugs dissolved in medium: chloroquine (CHQ, Sigma-Aldrich Co., St. Louis, MO), nanoparticles (NPs) at various concentrations, or a mixture of CHQ+NPs. The cells were allowed to incubate for time courses ranging from 1 hour to 12 days. For prolonged experiments, solutions were replaced at day 7 to ensure cell viability.

Lysosomal pH measurements were based on a protocol described in detail previously [5], [15], [16]. In brief, ARPE-19 cells were removed from the incubator and rinsed 3x with isotonic solution and then incubated with 5 µM LysoSensor Yellow/Blue DND-160 for 5 min; given the temperamental nature of the dye, this concentration was the minimum found to provide a consistent signal-to noise value. The dye exhibits a pH-dependent excitation at 340 nm and 380 nm and permits the ratiometric assessment of pH changes in acidic organelles independent of dye concentration. LysoSensor was removed from plate wells after ∼ 3 min and cells washed, followed by addition of either 100 µL control or pH calibration buffers. Most measurements were made 16–19 min after dye removal to minimize the slight alkalinizing actions of the dye. Lysosomal pH was determined from the ratio of light excited at 340 nm vs. 380 nm (>520 nm em); the measurement of control and experimental wells simultaneously in 96 well plates minimized changes attributable to the dye itself. Lysosomal pH values were calibrated in each plate at the same time as experimental levels and were determined by exposing cells to 10 µM H+/Na+ ionophore monensin and 20 µM H+/K+ ionophore nigericin in 20 MES, 110 KCl and 20 NaCl at pH 4.0–6.0 for 5 min. Fluorescence was measured with a Fluoroskan 96-well Plate Reader (Thermo Fisher). In spite of numerous steps taken to minimize variation (see [15]), some variation in absolute pH level did occur between plates measured at different days. However, normalization revealed that the relative differences between experimental and control pH values were constant.

### Assessment of Availability of Cathepsin D Active site with BODIPY FL-pepstatin A Probe

The availability of the cathepsin D active site was measured with the fluorescent probe BODIPY FL-pepstatin A (Invitrogen). The probe itself is synthesized by covalently conjugating the BODIPY (Boron dipyrromethene difluoride) fluorophore to pepstatin A, a potent and selective inhibitor of cathepsin D [26]. The probe binds directly to the active site of cathepsin D, providing a measure of access and potential cathepsin D activity. To localize the stain, ARPE-19 cells were grown on 12 mm cover glass and incubated in 10 µM BODIPY probe in DMEM/F12 for 30 min at 37°C in the dark, washed, and incubated in 5 µM LysoTracker Red DND-99 (Invitrogen) for 15 min. Cells were mounted and examined on the Eclipse microscope (Nikon). To quantify the availability of the cathepsin D active site, cells were grown to confluence on black-walled, clear-bottomed 96-well plates until confluent, and then incubated for 48 hrs. in either control culture medium, 10 µM CHQ in medium, or 10 µM CHQ +1 mg/mL NP3 in medium. After incubation, cells were washed followed by a 30 min incubation in 10 µM BODIPY probe at 37°C in the dark. Cells were washed again 3x and fluorescence compared with the Fluoroskan plate reader (Thermo Fisher), at 488 nm/527 nm (ex/em).

### Photoreceptor Outer Segment (POS) Preparation

The isolation of bovine photoreceptor outer segments was based on published protocols, with approval of the University of Pennsylvania IACUC [27]–[29]. Material was handled as previously published [15], [16]. Briefly, fresh bovine retinas were isolated under sterile conditions and stored at −80°C. Thawed retinas were agitated in 30% (w/w) buffered sucrose solution (containing 5 mM HEPES pH 7.4, 65 mM NaCl, 2 mM MgCl_2_) followed by centrifugation in a Sorval SS-34 rotor (7 min,700 rpm,4°C). The supernatant was diluted in two volumes of 10 mM HEPES pH 7.4 and further centrifuged (Sorval SS-34 rotor, 20 min, 17500 rpm, 4°C). The resulting pellet was then homogenized and layered on top of a discontinuous sucrose density gradient solution of 36, 32, and 26% sucrose (w/w); POS membranes were harvested from the 26%/32% sucrose solution interface. POS prepared this way were washed in 3 volumes of 0.02 M Tris buffer, pH 7.4 (Sorval SS-34 rotor, 10 min, 13000 rpm, 4°C). The pellet was resuspended in 2.5% (w/w) buffered sucrose solution (Na_2_HPO_4_ 10 mM, NaH_2_PO_4_ 10 mM, NaCl 1 mM) and stored at −80°C for later use.

### Autofluorescence Assay Using Flow Cytometry

ARPE-19 cells were grown to confluence in clear 6-well plates (BD Biosciences, Franklin Lakes, NJ). On day 0, cells in set one had culture medium removed and replaced with fresh medium (control), 10 µM chloroquine in medium, or 10 µM chloroquine +1 mg/ml NP3 in medium. Cells in set 2 had their medium removed and incubated with 2 ml POS in culture medium (10^6^ outer segments/ml) for 2 hours (pulse); the cells were washed thoroughly with IS to remove non internalized POS followed by 2 hours chase in medium. Subsequently, medium was removed and cells incubated for 20 hrs. with one of the following solutions: fresh medium (control), 10 µM chloroquine in medium, 10 µM chloroquine +1 mg/ml NP3, or 1 mg/ml NP3. This series of pulse/chase conditions was repeated for the subsequent 6 days. After 6 days the cells were washed, detached with 0.25% trypsin in EDTA (Invitrogen), and analyzed on a flow cytometer (FACS Calibur; BD Biosciences, Heidelberg, Germany) using the FITC channel (excitation laser wavelength, 488 nm; detection filter wavelength, 530 nm). The channel was gated to exclude cell debris and cell clusters.

### Opsin Immunoblotting

ARPE-19 cells were cultured on a 6-well plate until confluent. Cells were incubated with medium or 1×10^6^ POS/mL for 2 hrs at 37°C then washed 3x to remove non-internalized POS. Incubation in culture medium continued for a subsequent 2 hrs., after which 1 mg/mL NP3 were added and cells incubated as normal. After treating such every day for 7 days, cells were washed and processed for immunoblotting as published [30]. In brief, washed cells were lysed in 300 µl RIPA buffer (150 mM NaCl, 1.0% Triton X-100, 0.5% Na-Deoxycholate, 0.1% SDS, 50 mM Tris, pH 8.0 and protease inhibitor cocktail) and centrifuged at 13000 g for 10 min at 4°C. Protein concentrations were determined using the BCA kit (Pierce). Protein lysate (10 µg per lane) was loaded in sample buffer (2% SDS, 10% glycerol, 0.001% bromophenol blue, and 0.05 M Tris-HCl, pH 6.8), separated on a 4–15% gradient precast gel and transferred to a PVDF membrane. After blocking with SuperBlock buffer (Themoscientific, Rockford, IL) for 1 hr., the membrane was incubated with opsin antibody at 1∶1000 (#: SC-57432, Santa Cruz Biotechnology, Inc., Santa Cruz, CA) overnight at 4°C. The corresponding secondary antibody was developed with enhanced chemiluminescence (ECL, Amersham). Band intensity was detected and analyzed with ImageQuant LAS 4000 system (GE Healthcare Biosciences, Pittsburgh, PA). The blots were stripped and reprobed with rabbit monoclonal anti-GAPDH at 1∶1000 (#2118, Cell Signaling Technology) overnight at 4°C, and incubated with secondary antibody for 1hour at room temperature and processed as above.

### Statistical Analysis

Data are reported as mean ± SEM. Statistical analysis used a 1-way ANOVA with appropriate post-hoc test. Results with p<0.05 were considered significant.

## Results

### Characteristics of Nanoparticles

As the aim of this project was to acidify lysosomes using nanoparticles, nanoparticles were constructed using three different acidic polymers. As shown in Table 1, nanoparticle type 1 (NP1) was composed of PLGA Resomer® RG 502H and was the smallest molecular weight, with an acid number of 6 mg KOH/g. NP2 was composed of PLGA Resomer® RG 503H, intermediate in size and had an acid number of 3 mg KOH/g. NP3 was composed of PLA Resomer® R 203S and was the largest, with an acid number of ∼ 1 mg KOH/g. The particle size was 387.4–503.9 nm for blank NPs and 383.5–397.8 nm for Nile red loaded NPs. The polydispersity index ranged from 0.240–0.321 for blank NPs and 0.210–0.240 for Nile red loaded NPs.

### Nanoparticles Delivered to Lysosomes

Initial experiments were performed to determine whether nanoparticles were delivered to lysosomes. Nanoparticles were stained with Nile Red to facilitate localization. Figures 1A–D illustrate a typical experiment, where ARPE-19 cells incubated with NP2R-Nile Red. When cells were examined after a 24 hr. incubation in NP2R-Nile red, clustered red fluorescence was detected. This red fluorescence was largely observed in regions also stained with Lysotracker green, implying NP2R was localized to lysosomes (Figs 1A–D).

To determine the optimal conditions for nanoparticle delivery, the experiment was repeated using all three formulations but cells were examined for overlap between Lysotracker and nanoparticles after only one hour. Each nanoparticle formulation was tested at 0.25, 0.5, 1.0 and 2.0 mg/ml. To quantify the proportion of nanoparticles present in the lysosomes, overlap was calculated using Pearson’s coefficient. While 0.25 mg/ml gave lower levels, maximal overlap was observed with nanoparticles incubated at 1.0 mg/ml, with no further increase with increasing concentration. NP1R, NP2R and NP3R all gave similar results (Fig. 1E). As such, nanoparticles were given at 1 mg/ml in subsequent experiments.

**Figure 2:**
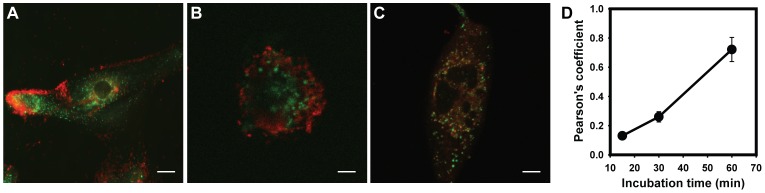
Rapid delivery of nanoparticles to lysosomes. A. Composite image of ARPE-19 cells with internalized NP3R nanoparticles (red, 1 mg/ml) and Lysotracker (green) after 15 min incubation. B. NP3R and lysosomes after 30 min incubation. C. NP3R and lysosomes after 1 hr. incubation. Bar  = 10 µM in panels A-C. D. Colocalization of NP3R nanoparticles with ARPE-19 cells as a function of time. Each point is the mean +/− standard deviation of the Pearson’s coefficient in one microscope field; n = 3. Error bars are present but too small to be detected for the 15 min point.

The preceding experiment indicated that nanoparticle delivery to the lysosome was largely complete within one hour. To determine the rate of internalization more accurately, cells were incubated with NP3R-Nile Red for 15, 30 and 60 min. Following this, cells were incubated for an additional 15 min with LysoTracker Green to visualize lysosomes, and the degree of colocalization determined. In cells exposed to NP3R-Nile Red for only 15 min, red fluorescence was largely localized on the cell periphery (Fig. 2A). Fluorescent red staining had extended in cells exposed to NP3R-Nile Red for 30 min (Fig. 2B), while red staining was observed throughout the cells after a 60 min exposure (Fig. 2C). Quantification supports these observations; only 13±3% of the NPs were colocalized in lysosomes after 15 min and 26±4% with lysosomes after 30 min. However, 72±8% of the NPs were in lysosomes after 60 min exposure (Fig. 2D). Of note, this level is remarkable close to that seen after 60 min in Figure 1D, supporting the observation that the majority of the nanoparticles are in the lysosomes after 60 min exposure.

### Nanoparticles Acidify Lysosomes

**Figure 3:**
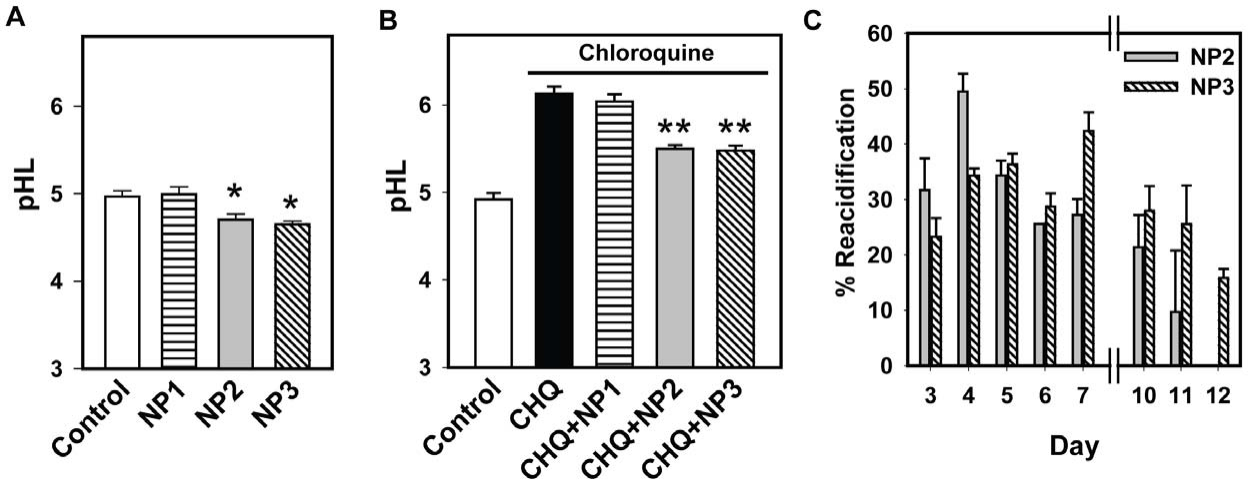
Nanoparticles lower lysosomal pH. A. While NP1 did not alter baseline levels of lysosomal pH (pHL), NP2 and NP3 acidified the lysosomes significantly. Lysosomal pH was measured 1 hr. after addition of nanoparticles. Here and throughout the figure, nanoparticles were given at 1 mg/ml. n = 8. * *p*<0.05 vs. control, ANOVA on ranks, Dunn’s posthoc test. B. Chloroquine (CHQ; 10 µM) raised the lysosomal pH. NP2 and NP3 significantly lowered lysosomal pH, while NP1 had little effect. Chloroquine and nanoparticles were applied concurrently 1 hr. before pH measurements. *p<0.001 vs. control, **p<0.001 vs. CHQ, ANOVA with Tukey post hoc test. n = 8. C. NP2 and NP3 induced sustained acidification of lysosomal pH in cells treated with 10 µM chloroquine. The acidification decreased with time but was still detected. Chloroquine and nanoparticles were added on day 1 and remained in the bath without a solution change. The effect of the nanoparticles was defined as their relative effectiveness at bringing lysosomal pH towards baseline; the absolute numbers did vary somewhat but this normalization accounted for such differences. % Reacidification  = 100*((CHQ-(CHQ+NP))/(CHQ-Control)). Data from 31 plates.

Lysosomal pH was measured to determine if acidic nanoparticles altered pH levels and to compare the efficacy of the different particles. Cells were incubated with non-fluorescent NP1, NP2 and NP3 for 1 hr. at 1 mg/ml, washed and loaded with the ratiometric lysosomal pH indicator Lysosensor Yellow/Blue. The effect of nanoparticles on baseline pH was measured first. NP1 had no effect on baseline pH. NP2 and NP3 both acidified the lysosomes, by 0.27 and 0.32 units respectively (Fig. 3A).

Recently we found that the magnitude of acidification can be greater in cells whose lysosomes are alkalinized by lysoosmotic agents [15]. Chloroquine has been well documented to elevate lysosomal pH through its actions as a tertiary amine [31], [32]. While the effect of chloroquine was dose dependent over this range (Supplemental Figure S1), we have previously found 10 µM produces relatively constant effects on lysosomal pH without leading to cell death. As such, the ability of nanoparticles to acidify lysosomes exposed to 10 µM chloroquine for 1 hr. was determined. The effect of NP1 was minimal, but NP2 and NP3 acidified cells by 0.62 and 0.64 units respectively (Fig. 3B).

To determine the duration of lysosomal acidification by nanoparticles, chloroquine was given on its own or in the presence of either NP2 or NP3 on day 0 and lysosomal pH was measured on various days afterwards. Overall, the mean lysosomal pH in control was 5.11±0.04 and in chloroquine was 5.75±0.006 (n = 32). Control cells showed a small 0.1 unit decrease in lysosomal pH from day 1 to 12 while cells treated with chloroquine had a 0.1 unit rise over this time. However, the variability between plates dictated that the effect of nanoparticles was best determined on a plate by plate basis, with normalization used to reduce variation found from multiple plates over numerous days. The effectiveness of nanoparticles was defined by the extent they could lower the pH in chloroquine treated cells with respect to that in untreated cells. To that end, both NP2 and NP3 proved capable of prolonged lysosomal acidification, lowering the pH in ARPE-19 lysosomes for over a week (Fig. 3C, see also Supplemental Figure S2). Thus acidic nanoparticles were capable of prolonged lysosomal acidification. Of note, cells undergoing various treatments appeared similar under gross examination, suggesting extended exposure to acidic nanoparticles or this concentration of chloroquine was not itself detrimental; this may be of interest with regard to the lethal effects of increased levels of chloroquine on these cells reported recently [33].

### Nanoparticles Improve Lysosome Function

**Figure 4:**
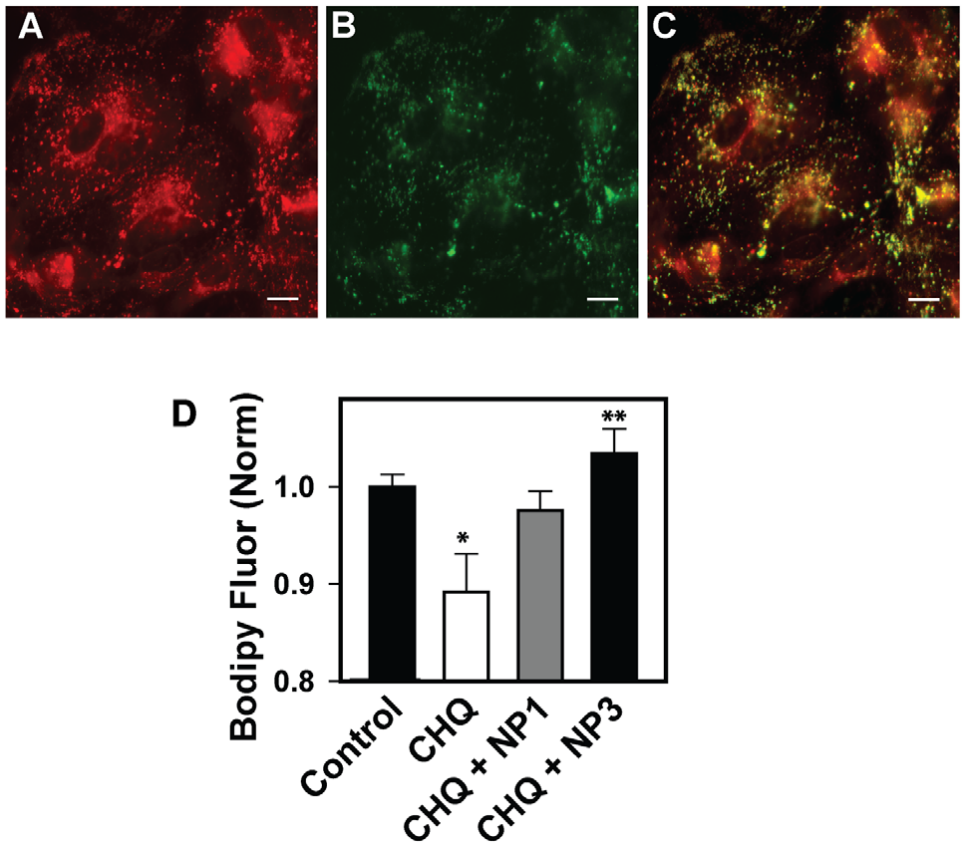
BODIPY FL-pepstatin A: Probing cathepsin D activity. A. Lysosomes of ARPE-19 cells as stained with LysoTracker Red B. BODIPY FL-pepstatin A staining. C. Composite image demonstrating colocalization of BODIPY fluorophore with RPE lysosomes D. CHQ administration raises the lysosomal pH, inactivating cathepsin D and hindering the binding of the BODIPY probe to the enzyme. This is seen quantitatively as a lowering in the amount of fluorescence (measured in arbitrary light units, ALU). Nanoparticles reverse this process, with NP3 significantly restoring cathepsin D activity. * p<0.05 vs. control, ** p<0.05 vs. CHQ; one-way ANOVA with Tukey post hoc test. n = 5 wells from 1 plate. Similar results seen in 2 plates.

While the absolute magnitude of the acidification induced by acidic nanoparticles was not huge, lysosomal enzymes are particularly sensitive to pH at this level [11]. As such, even a relatively modest acidification can have a substantial effect on the ability of the lysosomes to degrade material. Cathepsin D is a major protease in the lysosomes of RPE cells and contributes to the degradation of phagocytosed photoreceptor outer segments [34]. The availability of the cathepsin D active sight was monitored in situ with BODIPY FL-pepstatin A; the active site for cathepsin D is identified by the binding of fluorescently tagged substrate pepstatin A and availability is pH sensitive [11]. Co-incubation of ARPE-19 cells with BODIPY FL-pepstatin A demonstrated that the majority of the probe colocalized with LysoTracker Green (Fig. 4A–C). The amount of red probe was quantified to determine the effect of pH manipulation on the availability of the cathepsin D active site. As expected, chloroquine decreased the fluorescence readout, consistent with a decrease in potential cathepsin D activity with lysosomal alkalinization (Fig. 4D). Substrate binding to the cathepsin D active site was restored to baseline levels after treatment with NP3. These changes in the cathepsin D active site parallel the changes in lysosomal pH and suggest that the reacidification by nanoparticles may be sufficient to increase enzyme activity.

### Acidic Nanoparticles Increase Clearance of Photoreceptor Outer Segments

**Figure 5:**
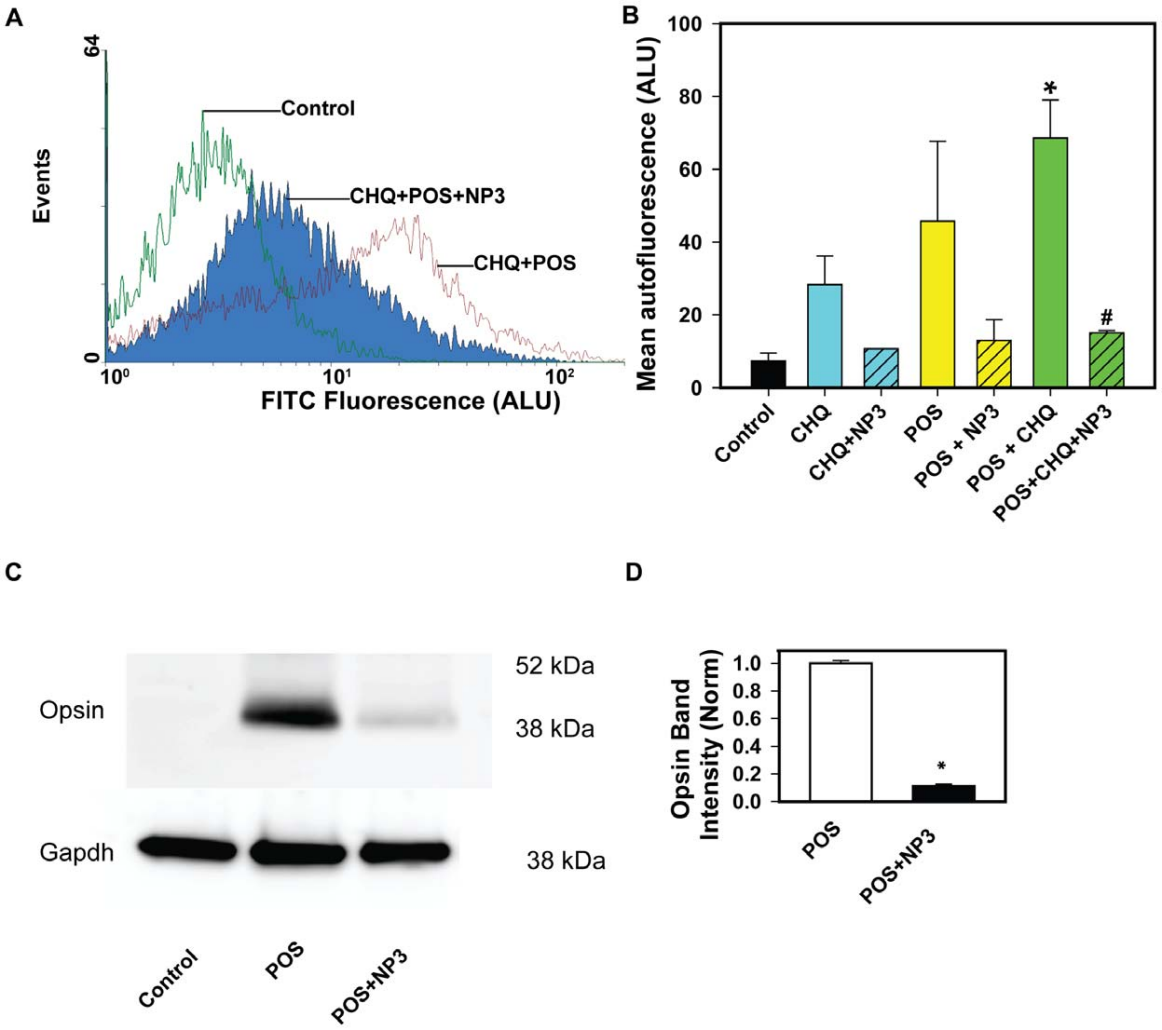
Nanoparticles reduce autofluorescence and opsin levels associated with ingestion of photoreceptor outer segments. A. Sample readout of the FACS analysis demonstrating treatment with NP3 greatly reduced the mean autofluorescence at 488 nm in RPE cells treated with chloroquine (CHQ) and phagocytosed photoreceptor outer segments (POS). B. Nanoparticles reduced the autofluorescence in ARPE-19 cells given POS, CHQ, and or POS+CHQ. Bars represent the mean ± SEM of autofluorescence detected at 488 nm. * p<0.05 vs. control; # p<0.05 vs. POS+CHQ. ANOVA. C. Immunoblot for opsin detected in ARPE-19 cells in the absence of photoreceptor outer segments (Control), after exposure to outer segments over 7 days (POS), and with a delayed addition of PLA NP3 after each outer segment feeding (POS+NP3). The blot was at the predicted size of ∼40 kDa. GAPDH binding of the blot is demonstrated below. D. Quantitation of opsin levels in immunoblots. Levels were first controlled for GAPDH staining, and then normalized to the mean POS value in each blot to control for variation. * p<0.001, n = 4.

RPE lysosomes are required to degrade engulfed photoreceptors through heterophagy and extraneous internal material through autophagy. Elevation of lysosomal pH is predicted to be detrimental to both types of degradation, and acidic nanoparticles have the potential to improve both. To examine the impact of nanoparticles on heterophagy, ARPE-19 cells were fed photoreceptor outer segments for 2 hrs., followed by a two hour wash to allow for internalization. After this interval, half of the wells were exposed to NP3 for 20 hrs. The cycle was repeated for 5 more days (6 exposure cycles in total), after which cells were detached and autofluorescence at 488 nm was determined using FACS analysis. NP3 was used as it demonstrated the greatest promise for sustained decrease of lysosomal pH. Treatment with photoreceptor outer segments increased autofluorescence 4 fold, but subsequent exposure to NP3 reduced this autofluorescence (Supplemental Figure S3). Interestingly, exposure to chloroquine alone increased the autofluorescence four-fold, indicating a decreased processing in material of cellular origin. However, inclusion of NP3 in the treatment substantially reduced the autofluorescence. Finally, to test if the effects were additive, chloroquine or chloroquine+NP3 was added to the cells 2 hrs. after washing off the outer segments for 6 days. Together, chloroquine and photoreceptors increased autofluorescence ten-fold, suggesting the effects were, at a minimum, additive (Fig. 5A). However, NP3 greatly reduced the autofluorescence seen when both challenges were added. The mean changes in total autofluorescence at 488 nm induced by combinations of POS, chloroquine and NP3 are presented in Fig. 5B. In each of the flow cytometry experiments conducted, NP3 consistently reduced autofluorescence.

The ability of PLA NP3 to enhance outer segment clearance was tested directly by quantifying the amount of opsin present in the cells with the immunoblotting technique. Confluent ARPE-19 cells were exposed to photoreceptor outer segments with or without PLA NP3 nanoparticles using the pulse chase protocol described above. No opsin was detected in cells not exposed to outer segments, although the band intensity increased in cells challenged with outer segments, consistent with the increased autofluorescence above (Fig. 5C). However, treatment with NP3 decreased the band intensity substantially. Quantification indicated that NP3 treatment reduced mean opsin levels in cells by over 90% (Fig. 5D). This supports the concept that acidifying lysosomes with acidic nanoparticles can enhance the degradation and clearance of photoreceptor outer segments by RPE cells.

## Discussion

**Figure 6:**
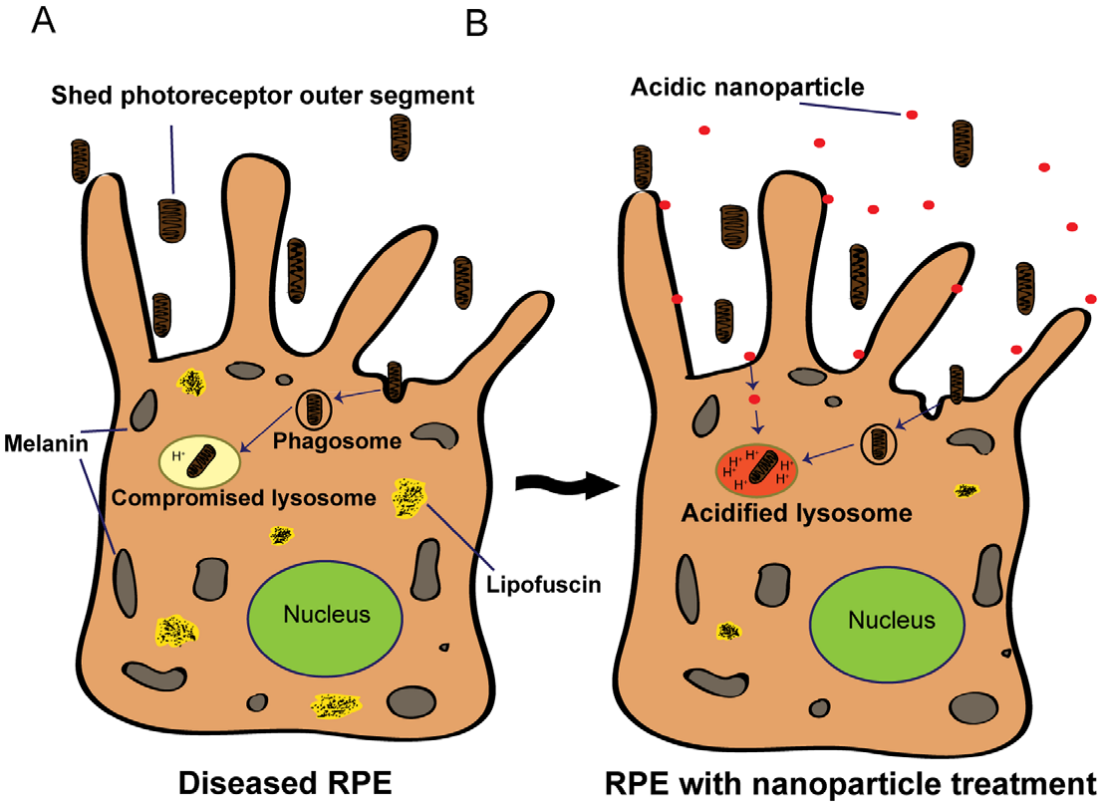
Model of enhanced photoreceptor degradation. A. RPE cells with compromised lysosomes cannot sufficiently degrade photoreceptor outer segments. The undigested material accumulates inside the cell as autofluorescent lipofuscin. B. After treatment with acidic nanoparticles, RPE lysosomes are more capable of breaking down the POS. The end result is a substantial decrease in undigested debris and lipofuscin.

Nanoparticles have great promise for drug delivery, but their delivery to the correct target is critical. This study turns their propensity for lysosomal accumulation into an advantage, and demonstrates that acidic nanoparticles can lower the pH of compromised lysosomes to improve degradative function. Nanoparticles localized to lysosomes over the course of one hour, with a saturating concentration of 1 mg/ml (Figs 1–2). Acidic particles seemed to function in lysosomes; pH was significantly reduced one hour after treatment, with acidification remaining after 12 days (Fig 3). Acidic nanoparticles restored the availability of the cathepsin D active site (Fig. 4), and greatly increased the clearance of autofluorescent material and opsin (Fig. 5). Together, these observations lead us to propose a model whereby the ingested acidic nanoparticles can reduce the accumulation of partially degraded autofluorescence material in RPE cells (Fig. 6). Given the critical role that lysosomal enzymes play in general cellular maintenance, the potential for acidic nanoparticles to improve degradative function has implications for a broad range of cell types.

### Rapid and Sustained Delivery of Nanoparticle to Lysosome

The results above imply that acidic nanoparticles are delivered rapidly to lysosomes and remain active for at least a week. Measurement of lysosomal pH taken one hour after adding acidic nanoparticles to the bath demonstrated a clear acidification. Functional evidence is consistent with microscopic data confirming the majority of nanoparticles localized to lysosomes 60 min after addition. Although the pathways used to these deliver nanoparticles are unknown, it is of interest that the colocalization rate was not affected by the acid number of the material used to construct the nanoparticle nor the molecular weight. It was recently reported that most nanoparticles given to macrophages ended up in the lysosome regardless of the endocytotic pathways used [35], consistent with our findings. It is also intriguing that the particle with the lowest acid number was most effective at lowering lysosomal pH.

While the lysotracker dye labels acidic organelles, and nanoparticles are acidic, several observations testify to the independence of the staining. First, if Lysotracker just went to the nanoparticles, lower concentrations of nanoparticles would have more than enough Lysotracker and display increased colocalization; Figure 1E shows the opposite occurs. Likewise, if Lysotracker was just labeling nanoparticles, then rates of colocalization would be instantaneous, and not delayed, as illustrated in Figure 2D. A recent study using MDCK and Caco-2 cells exposed to coated microbeads suggested that internalization required 23–32 min, phagosomal acidification took 3–4 min and fusion of the phagosome to endosome/lysosomes was complete within 74–120 min [36]. The similar time course for colocalization of Lysotracker and nanoparticles strongly suggests delivery of the nanoparticles into the lysosomes. Third, the degree of costaining was not affected by the different acid number of NP1-3, suggesting the fluorescence was not a direct reflection of acidity but a more complex reaction. Finally, there was a clear increase in degradative activity of lysosomal enzymes in the presence of the nanoparticles. Together, these observations imply that nanoparticles are present in the lysosomes.

Although endocytosis itself may initiate a series of changes to lysosomes, it is unlikely that nanoparticle internalization can itself lower lysosomal pH as all three nanoparticle formulations were delivered to the lysosome but only two of them lowered lysosomal pH. It is unclear why the PLA-based NP3 was more effective at restoring the cathepsin D active site than NP2 given their similar effects on pH, although the enhanced performance of NP3 over the long term suggests it would be a better drug overall.

### Functional Restoration by Acidic Nanoparticles

While the absolute decrease on pH values induced by acidic nanoparticles was relatively small, this shift is expected to have an impact on degradation because of the sharp relationship between pH and enzyme activity over this range. For example, the activity of cathepsin D increases 3 fold when pH falls from 5.0 to 4.5 [11] and acid lipase activity more than doubles when pH falls from 5.2 to 4.5 [10]. The magnitude of the acidification induced by nanoparticles was larger in compromised lysosomes with elevated pH than at baseline levels. This is desirable with regards to treatment, as it will enable the particles to preferentially target the more disturbed organelles in a mixed population.

The effectiveness of the acid nanoparticles was confirmed by the increase in binding of Bodipy-pepstatin A to the cathepsin D active site, by the decrease in opsin, and by the decrease in autofluorescence. The fluorescent Bodipy-pepstatin-A binds to the active site of cathepsin D, so the enhanced signal observed after treatment with acidic nanoparticles could reflect an increase in the pH-sensitive maturation of cathepsin D [37], a direct effect of pH on active site availability [11], or both. In rat RPE cells, opsin was shown to disappear from RPE phagosomes that costained for cathepsin D, but blockage of the vHATPase with bafilomycin prevented this opsin degradation [38]. The ability of NP3 to decrease immunoblotting for opsin is consistent with improved degradation by cathepsin D once acidity is restored.

The reduced lipofuscin-like autofluorescence observed after nanoparticle treatment further supports the functional effectiveness of the acid nanoparticles. The increased autofluorescence detected in cells treated just with chloroquine is consistent with an incomplete degradation of autophagic material following elevated lysosomal pH, although this needs confirmation. The increased autofluorescence in cells treated with photoreceptor outer segments is consistent with a retinoid component and is supported by changes in cellular opsin levels. The ability of acid nanoparticles to greatly reduce both forms of autofluorescence may have important implications for the treatment of macular degeneration, as the accumulation of autofluorescent lipofuscin has been associated by some with disease progression [12]–[14]. The ability of NP3 treatment to significantly reduce the levels of cellular opsin detected in immunoblots provides direct evidence that the nanoparticles enhance degradation and clearance of photoreceptor outer segments. In this regard, the sustained acidification observed following treatment in Figure 3 may be of benefit. Nanoparticles composed of PLA remained in RPE cells without being degraded for up to 4 months following a single injection [39], consistent with the slower degradation of these particles in lower pH [40]. The degradation of PLA into non-toxic lactic acid suggests their appropriateness as a chronic treatment, although it remains to be determined if acid nanoparticles can lead to a sustained improvement of opsin degradation in vivo.

## Supporting Information

Figure S1 **Increasing concentrations of chloroquine lead to proportional increases in lysosomal pH in ARPE-19 cells.** Addition of 1, 10 or 20 µM chloroquine let to increasing magnitudes of lysosomal alkalinization when examined 15 (upper panel) or 60 (lower panel) min after addition.
(EPS)

Figure S2 **Long term acidification of ARPE-19 cells by nanoparticles.** These figures were meant to show the long term effects of the nanoparticles ranging from 1–12 days. Of note is the observance that NP1(A) after 1 day never acidified the lysosomes, explaining why the % acidification is always negative. NP2 (B) and NP3 (C) were much more promising, with maximum acidification in the range of 50%. NP2 seemed to peak earlier and acidification dropped rather predictably over the 12 days, while NP3 seemed to slowly peak at day 7 days and then drop.
(TIF)

Figure S3 **FACS histograms of nanoparticles reducing autofluorescence in ARPE-19 cells given outer segments OR CHQ.** ARPE-19 cells were fed bovine POS for 2 hours, washed, and two hours were allowed for outer segment delivery to the lysosomes. At this point, nanoparticles were added to the cells. Adding the particles after the two hour interval ensured effects were restricted to outer segment digestion and did not alter binding or phagocytosis. This two stage treatment was repeated every day for multiple days. Cells were then dissociated and the autofluorescence at 488/520 (ex/em) was determined using flow cytometry. Nanoparticle 3 lowered the lipofuscin-like autofluorescence that the cells acquired from digesting POS. NP3 lowered the fluorescence to almost baseline levels.
(TIF)
