# Supplementary figures and images for: Acidic Nanoparticles Are Trafficked to Lysosomes and Restore an Acidic Lysosomal pH and Degradative Function to Compromised ARPE-19 Cells
Source: PLoS One. 2012 Dec 18;7(12):e49635. doi: 10.1371/journal.pone.0049635 (PMC3525582; doi:10.1371/journal.pone.0049635)

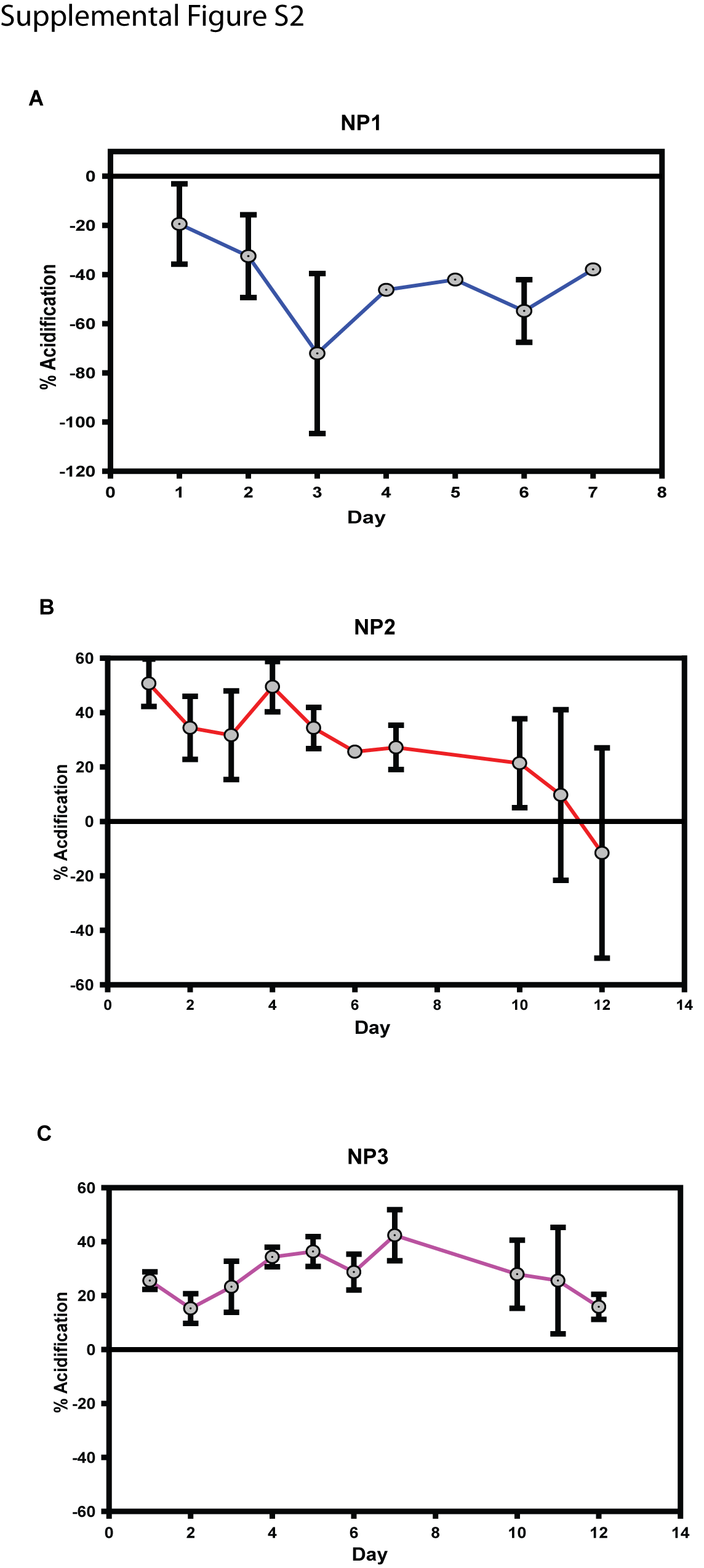

Supplement: Figure S2 — Long term acidification of ARPE-19 cells by nanoparticles. These figures were meant to show the long term effects of the nanoparticles ranging from 1–12 days. Of note is the observance that NP1(A) after 1 day never acidified the lysosomes, explaining why the % acidification is always negative. NP2 (B) and NP3 (C) were much more promising, with maximum acidification in the range of 50%. NP2 seemed to peak earlier and acidification dropped rather predictably over the 12 days, while NP3 seemed to slowly peak at day 7 days and then drop. (TIF) [file pone.0049635.s002.tif]

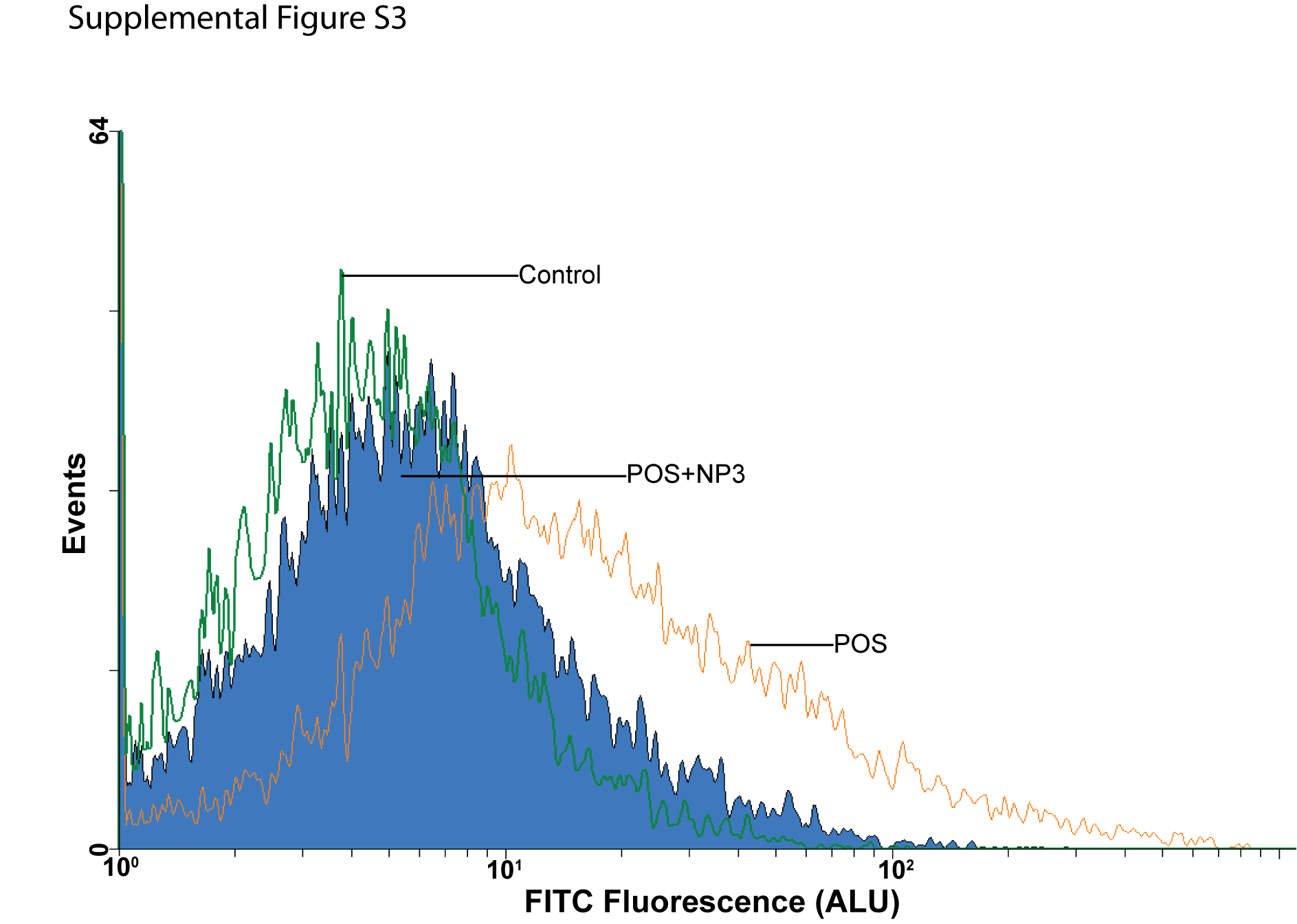

Supplement: Figure S3 — FACS histograms of nanoparticles reducing autofluorescence in ARPE-19 cells given outer segments OR CHQ. ARPE-19 cells were fed bovine POS for 2 hours, washed, and two hours were allowed for outer segment delivery to the lysosomes. At this point, nanoparticles were added to the cells. Adding the particles after the two hour interval ensured effects were restricted to outer segment digestion and did not alter binding or phagocytosis. This two stage treatment was repeated every day for multiple days. Cells were then dissociated and the autofluorescence at 488/520 (ex/em) was determined using flow cytometry. Nanoparticle 3 lowered the lipofuscin-like autofluorescence that the cells acquired from digesting POS. NP3 lowered the fluorescence to almost baseline levels. (TIF) [file pone.0049635.s003.tif]
